# Relative peripheral refraction with accommodation in 6- to 11-year-olds: baseline findings from the Stockholm Myopia Study

**DOI:** 10.1364/BOE.559666

**Published:** 2025-05-30

**Authors:** Charlie Börjeson, Anna-Caisa Söderberg, Anna Lindskoog Pettersson, Peter Unsbo, Linda Lundström

**Affiliations:** 1 Department of Applied Physics, KTH Royal Institute of Technology, Sweden; 2 Department of Health Sciences, Mid Sweden University, Sweden; 3 Department of Clinical Neuroscience, Karolinska Institutet, Sweden

## Abstract

This study compares image quality on the peripheral retina for far and near vision in schoolchildren. Biometric data and simultaneous foveal and peripheral ( $\pm 25^{\circ }$ horizontal field) wavefront data for two levels of accommodation (0.22 D and 5 D) were collected from 31 children aged 6 to 11 years. Relative peripheral refraction (RPR) was found to be larger and more negative in the nasal visual field than in the temporal. This difference increased with accommodation. Furthermore, correlations between image quality and biometric parameters were investigated. The results highlight the importance of peripheral image quality during near work for myopia research. The data presented also form the baseline measurements of the Stockholm Myopia Study, which is a longitudinal pilot study on ocular growth and peripheral image quality in schoolchildren in Stockholm, Sweden.

## 1. Introduction

Myopia (nearsightedness) is a growing global health problem, with 50% of the world’s population estimated to be myopic by the year 2050 [1,2]. This “myopia boom” is most prominent in East Asia [3,4], with cities like Seoul having a myopia prevalence of 96.5% in the 19-year-old male population [5]. Myopia is also increasing in other regions of the world, although the levels are not yet as high as in East Asia [2,6]. Unless this trend is reversed, myopia will be one of the leading causes of blindness in the world by 2050 [2], due to a substantially increased risk of vision-threatening ocular diseases with high myopia [1].

To combat the growing global myopia prevalence, both academia and industry have been searching for ways to prevent or reduce myopic growth in children. In recent years, peripheral image quality has gained more focus due to its apparent connection with myopia development. Animal studies have shown that induced blur in the periphery affects eye growth [7,8], with induced hypermetropic peripheral defocus triggering growth, and induced myopic peripheral defocus preventing growth [9–11]. This has, in turn, led to the design of optical myopia control therapies, which often aim to induce some negative (myopic) peripheral defocus [12–14]. However, it has come into question whether relative peripheral refraction (RPR) actually affects myopia development in humans [15], or whether other optical effects in the periphery play a role [16,17]. Another problem is that RPR is often measured under cycloplegia, which limits the generalizability of the results, since cycloplegia itself can alter the RPR [18].

Additionally, near work has been linked to myopia progression [19–21]. Possible mechanisms for this correlation could, for instance, be lag of accommodation, which places the foveal image behind the retina, or changes in the peripheral refraction and image quality [22]. Since peripheral image quality itself seems to be connected with myopia development, any such changes with accommodation could be a clue to understanding myopia. However, to our knowledge, studies on accommodation-induced changes in peripheral image quality have only been conducted on adults so far.

There is clearly a need for more knowledge on the peripheral image quality in children, especially during near work, and its longitudinal implications for myopic development. For this reason, the Stockholm Myopia Study was started in 2023. It is a longitudinal pilot study aiming to follow children from the Stockholm region in Sweden over 5+ years. The novelty of this study is the investigation of peripheral image quality during accommodation, using a unique open-field dual-angle wavefront aberrometer, in a pediatric setting. In the Stockholm Myopia Study, we measure uncycloplegic RPR for both far and near targets, as well as ocular biometry. In the short-term, this will give us insight into the effect of accommodation on RPR in children. In the future, long-term, it will also shed light on the optics’ role in myopia development.

The current article presents the *baseline* findings from the Stockholm Myopia Study, where measurements were conducted on 33 children aged 6–11 years. Of particular focus is the RPR, which was measured using the dual-angle wavefront aberrometer. This instrument monitors the accommodation with a foveal wavefront sensor, while a peripheral sensor simultaneously measures the wavefront aberrations in the $25^{\circ }$ nasal or temporal visual field [23]. This allows us to investigate the effect of near work on peripheral image quality and RPR during normal binocular viewing conditions, even in the presence of accommodative lead or lag. Additionally, ocular biometry results are presented to show the representation of the study group, and to explore potential biometric correlations within the group.

## 2. Methods

The Stockholm Myopia Study is a pilot study investigating peripheral image quality during near work in children. In the present article, the baseline results are presented. In the coming years, the children will be followed during their emmetropization/myopia development, allowing investigation of links between ocular growth and peripheral image quality.

The novelty of the Stockholm Myopia Study is the investigation of peripheral image quality in children during accommodation, using an open-field dual-angle wavefront aberrometer. This instrument enables accurate measurement of RPR, even in the presence of accommodative lead or lag, since the foveal and peripheral visual fields are measured simultaneously.

### 2.1. Protocol and measurements

The study was conducted in accordance with the Declaration of Helsinki, and was approved by the Swedish Ethical Review Authority (Reference No. 2023-01477-01). Children were recruited from schools, optometrists and eye clinics in the Stockholm area. The aim was to obtain a group of children with good eye health and normal refractive variations, with most being non-myopic at baseline (but with some potentially turning myopic in the coming years). The inclusion criteria were: age 6–15 years at baseline (primary target age 6–9 years); normal binocular distance visual acuity (with correction if needed) for their age ( ≥
0.8 in decimal visual acuity for ages 6–9 years, ≥
1.0 for ages 10–15 years); and normal accommodation and stereopsis. The exclusion criteria were: ocular surgery; medical condition or medication affecting the eyes; premature birth (< week 37); high myopia ( <−
4.75 D); high astigmatism ( <−
2.50 D); anisometropia ( ≥
1.50 D); heterotropia; and, history of myopia control.

Informed consent was obtained from the parents and subjects prior to the measurements. The first part of the measurement protocol consisted of tests to ensure normal visual function of the subjects. The tests included:

• Visual inspection of the eyes
• Visual acuity (monocular and binocular at far and near)
• Cover test (far and near)
• Lang stereo test II
• Accommodation range (monocular and binocular, repeated thrice)
• Convergence near point (repeated thrice)

**Fig. 1.**
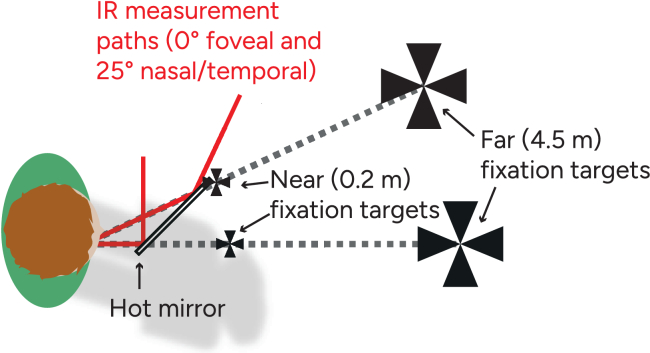
Illustration of the measurement setup (top view). Infra-red (IR) light is sent to the right eye and is then collected in two measurements paths (one foveal, one peripheral) for wavefront analysis. A hot mirror is used to allow a binocular field-of-view for the subject. Maltese crosses are used as fixation targets, at two different distances. If the subject looks at one of the left targets (from the subject’s point of view), the foveal and temporal visual fields are measured. Conversely, if the subject looks at one of the right targets, the foveal and nasal visual fields are measured.

The second part of the protocol consisted of simultaneous foveal and peripheral wavefront measurements of the right eye with a dual-angle wavefront aberrometer (described in [23]). This instrument has an open-field setup which allows for binocular viewing of an accommodation target. Since we wanted to measure the every-day image quality that the eye experiences, both for distance viewing and near work, no cycloplegia was used, and subjects who wore habitual correction were instructed to keep their correction on during wavefront measurements. We used two different accommodation targets, Maltese crosses with $2^{\circ }$ visual angle, located at 4.5 m (0.22 D) and 0.2 m (5 D) from the eye (see Fig. 1). To obtain both nasal and temporal peripheral measurements for the two accommodation distances, four sets of measurements were performed:

• Foveal and $25^{\circ }$ nasal at 4.5 m target distance.
• Foveal and $25^{\circ }$ temporal at 4.5 m target distance.
• Foveal and $25^{\circ }$ nasal at 0.2 m target distance.
• Foveal and $25^{\circ }$ temporal at 0.2 m target distance.

When measurements were performed, the light in the room was dimmed. Wavefronts were captured continuously (frame rate of ≈
6 Hz per sensor) for a couple of seconds, repeated at least thrice to account for sudden head movements, blinks, or other issues. Subjects were instructed to keep fixating on the target during measurements, and it was continuously monitored by the instructors that they did so, both by direct observation of their eye movements and by observation of the live spotfield pattens. The subjects were allowed brakes between the measurements to reduce eye strain and improve compliance.

After the wavefront measurements, cycloplegia was induced by instilling 1–2 drops of 1% cyclopentolate (depending on eye color and administration method). If the expected cycloplegic effect was not achieved within 15 minutes, an additional dose of cyclopentolate was administered.

Finally, after achieving cycloplegia, the third and final part of the protocol proceeded. Biometric measurements were obtained using the Eyestar 900 (Haag-Streit AG, Switzerland), which employs swept-source optical coherence tomography (SS-OCT). This device has demonstrated high repeatability across all measurements and good agreement with other instruments utilizing the same technology [24,25]. Cycloplegic autorefraction measurements were then conducted using the Wave Analyzer Medica 700 (Essilor Instruments, USA), which operates using Hartmann-Shack wavefront sensing [26]. Three measurements were performed for each subject, recording the 3 mm pupil aperture values. The spherical equivalent refraction (SER) was calculated as the mean of the three recorded values.

### 2.2. Data analysis

From the biometric measurements with the Eyestar 900, corneal radius of curvature was calculated as $r=(\frac{n-1}{K_{1}}+\frac{n-1}{K_{2}})/2$ with n=1.3375 , where $K_{1}$ and $K_{2}$ are the corneal optical powers in the flattest and steepest meridian. To assess the impact of lens tilt on horizontal RPR, the horizontal equivalent lens tilt (LTX) was calculated from the lens tilt axial angle (LTA) and the lens tilt rotational angle (LTR) as LTX=−
LTA⋅
cos⁡
(LTR) . Additionally, total (= radial) lens decentration (LDR) was calculated from the horizontal and vertical lens decentrations (LDX and LDY, respectively).

To investigate correlations between different biometric parameters, correlation tests were performed between axial length (AL), SER, age, anterior chamber depth (ACD), lens thickness (LT), corneal radius of curvature (CR), AL/CR ratio, LDR, and LTA, using Spearman correlation.

To account for the large number of repeated correlation tests, Bonferroni correction was applied to yield an adjusted α
 -value. The adjusted α
 -value was 0.05/n , where n was the number of comparisons. This ensured that there would only be a 5% chance of falsely significant results.

Only values from the right eye were used in the biometric analysis, to be able to correlate the findings with those from the dual-angle aberrometer.

Before processing the captured wavefronts, manual screening of the raw spotfield images from the wavefront sensors was performed. Images were excluded from analysis if there were strong corneal reflections, if the eye was misaligned, if a large part of the eyelids obscured the pupil, if the eye moved quickly, or if the image was degraded in some other way, for example by obscuring hair. The remaining images were processed in MATLAB, yielding modal reconstructions with Zernike polynomials. Chromatic aberration was compensated for by converting reconstructed Zernike coefficients from 830 nm (measurement wavelength) to 550 nm. Refraction, both foveally and peripherally, was then calculated using $2^{\text{nd}}$ -order Zernike coefficients. Moreover, refraction was calculated for three pupil conditions: natural pupil size, scaled to 4 mm diameter, and scaled to 3 mm diameter.

As the benefit of the dual-angle instrument is the simultaneous acquisition of foveal and peripheral wavefronts, any wavefronts that did not have a matching foveal/peripheral wavefront were not included in the analysis. Furthermore, the calculated mean spheres from each wavefront were manually assessed to identify possible errors in the reconstruction or large deviations in the accommodation. Any identified errors or deviations were also excluded from analysis.

RPR was calculated from the remaining wavefront pairs as the difference in refraction (mean sphere) between the periphery and fovea: $\text{RPR}=M_{peripheral}-M_{foveal}$ . Median values of RPR for nasal far, nasal near, temporal far, and temporal near were calculated for each subject. Additionally, the difference in RPR between near and far, i.e. the RPR change with accommodation, was calculated: ${\text{RPR}}_{change}={\text{RPR}}_{near}-{\text{RPR}}_{far}$ . The significance of this change with accommodation was tested with the Wilcoxon signed rank test. Bonferroni correction was used to account for the repeated RPR tests. Furthermore, the accommodative response change between far and near was calculated as $M_{fov,far}-M_{fov,near}$ .

As a further measure of peripheral image quality, Strehl ratios for the nasal and temporal visual fields were calculated from the wavefront measurements for 3 mm pupil diameters. Defocus was compensated for, using two different methods: either by subtracting the foveal mean sphere refraction, or by subtracting the accommodation target vergence. This produced the Strehl ratio for an object either at the accommodation distance, or at the target distance, respectively. If a subject had no accommodation lead or lag, these two methods would yield the same result. The Stiles-Crawford effect was taken into account when calculating the Strehl ratios, using the formula described by Atchison et al. [27]. Strehl ratios were calculated for both the nasal and temporal visual fields, as well as for near and far.

To assess the relationship between peripheral image quality and refractive development, the calculated Strehl ratios and RPR parameters (for 3 mm pupils) were compared to axial length, age and SER with correlation analysis, using Spearman correlation. Additionally, RPR at far was compared to LDX and LTX, to assess potential influences of lens position on RPR. Only the measurements at far were used for this latter analysis, as the biometric measurements of LDX and LTX were performed under cycloplegia. Bonferroni correction was used to account for the large number of repeated comparisons.

## 3. Results

33 children (20 female, 13 male) aged 6–11 years were included in the present study, forming the baseline measurements of the Stockholm Myopia Study. The participants were all residents of Stockholm, representing a diverse range of ethnic backgrounds, including 18 Caucasian, 2 Middle Eastern, 2 Asian, and 11 mixed (with parents of different ethnic origins). Ages ranged from 6 to 11 years, with five 6-year-olds, eleven 7-year-olds, seven 8-year-olds, eight 9-year-olds, and two 11-year-olds, with a mean age of 8.2 ( ±
 1.3) years. Measurements were performed between September 2023 and June 2024. For two of the children, wavefront data is incomplete due to a large amount of failed wavefront captures, leaving 31 children for whom we have complete data.

### 3.1. Biometry and autorefraction

Individual results of the biometric measurements of the right eye for our 33 subjects are reported in Table 1. The subjects are grouped by age and sorted by cycloplegic SER. As can be seen, the mean SER of the right eye was +0.61 ( ±
1.14 ) D, and the mean axial length (AL) was 22.88 ( ±
0.79 ) mm. Five of the children were myopic (SER ≤
−
0.5 D) whilst the rest had SER between −
0.5 D and +2.29 D.

**Table 1.** Individual biometric baseline data – right eye. Standard deviations for mean values are given within parentheses. Subjects are sorted by age and SER. Abbreviations: SER = spherical equivalent refraction, AL = axial length, LTA = lens tilt axial angle (angle between lens axis and corneal vertex normal), LTR = lens tilt rotational angle (direction of the tilt), LDX = lens decentration x-axis, LDY = lens decentration y-axis, LT = lens thickness, ACD = anterior chamber depth (including the cornea), CR = corneal radius of curvature.

**Subject**	**Age [years]**	**SER [D]**	**AL [mm]**	**LTA [°]**	**LTR [°]**	**LDX [mm]**	**LDY [mm]**	**LT [mm]**	**ACD [mm]**	**CR [mm]**
S09	6.7	+1.88	21.67	4.65	203	-0.06	+0.13	3.47	3.61	7.52
S32	6.0	+1.21	22.19	4.20	193	-0.05	+0.08	3.72	3.48	7.65
S23	6.1	+0.17	22.68	4.88	200	-0.14	+0.21	3.26	3.69	7.56
S27	6.3	+0.17	22.54	3.88	203	-0.03	+0.02	3.36	3.77	7.58
S31^*a*^	6.7	-3.00	22.70	3.04	222	-0.01	+0.08	3.67	3.31	7.63
**Mean 6 y/o**	**6.4 (±0.3)**	**+0.08 (±1.87)**	**22.36 (±0.43)**	**4.13 (±0.72)**	**204 (±11)**	**-0.06 (±0.05)**	**+0.10 (±0.07)**	**3.50 (±0.20)**	**3.57 (±0.29)**	**7.59 (±0.05)**
S16	7.8	+1.58	21.72	4.41	202	-0.14	+0.11	3.40	3.44	7.46
S06	7.1	+1.38	22.12	2.99	188	-0.01	+0.06	3.37	3.81	7.48
S24	7.1	+1.33	22.41	2.96	207	±0.00	+0.11	3.53	3.70	7.62
S04	7.2	+1.17	22.53	4.67	193	-0.02	+0.13	3.21	3.90	7.58
S07	7.1	+1.04	22.23	4.39	211	-0.02	+0.03	3.49	3.80	7.67
S30	7.6	+0.96	22.48	3.66	200	-0.06	+0.10	3.27	3.70	7.59
S01	7.7	+0.50	22.42	4.96	201	-0.12	+0.04	3.58	3.58	7.65
S02	7.4	+0.42	22.81	3.78	201	-0.14	-0.03	3.36	3.51	7.87
S10	7.5	+0.29	22.70	4.73	193	-0.04	±0.00	3.46	3.47	8.03
S17	7.8	-0.17	22.16	2.53	182	+0.01	+0.11	3.54	3.75	7.41
S34	7.0	-0.75	23.61	5.27	189	+0.02	+0.11	3.34	4.15	7.65
**Mean 7 y/o**	**7.4 (±0.3)**	**+0.70 (±0.72)**	**22.47 (±0.46)**	**4.03 (±0.91)**	**197 (±9)**	**-0.05 (±0.06)**	**+0.07 (±0.05)**	**3.41 (±0.12)**	**3.71 (±0.21)**	**7.64 (±0.18)**
S37	8.9	+1.38	23.68	3.92	196	-0.06	+0.10	3.35	3.60	8.14
S14	8.7	+1.29	22.15	4.57	196	+0.01	+0.18	3.36	3.98	7.36
S08	8.4	+1.25	22.75	1.69	233	-0.03	-0.01	3.49	2.99	7.99
S21	8.9	+1.13	24.07	4.81	203	-0.16	+0.07	3.50	3.67	8.40
S12	8.6	+1.00	22.70	5.51	184	-0.12	+0.21	3.40	3.76	7.77
S36	8.6	+0.17	24.08	4.35	179	-0.07	+0.23	3.32	3.85	8.06
S33	8.0	-0.79	23.59	3.71	203	-0.01	-0.01	3.48	4.11	7.62
**Mean 8 y/o**	**8.6 (±0.3)**	**+0.77 (±0.80**	**23.29 (±0.75)**	**4.08 (±1.21)**	**199 (±18)**	**-0.06 (±0.06)**	**+0.11 (±0.10)**	**3.41 (±0.07)**	**3.71 (±0.36)**	**7.91 (±0.35)**
S29^*a*^	9.1	+2.29	22.17	5.26	196	-0.16	+0.16	3.45	3.48	7.86
S19	9.4	+1.92	23.43	4.24	186	-0.10	+0.14	3.17	3.78	8.01
S38	9.3	+1.63	22.30	4.96	189	-0.01	+0.15	3.35	3.94	7.53
S20	9.0	+0.79	23.69	3.61	201	+0.01	+0.09	3.52	3.72	7.98
S35	9.4	+0.71	22.60	2.91	188	+0.02	+0.13	3.23	4.12	7.42
S26	9.8	+0.54	23.99	2.62	191	+0.07	+0.11	3.10	4.07	7.74
S25	9.8	+0.50	24.92	3.27	204	-0.08	+0.09	3.38	3.86	8.27
S18^*a*^	9.0	-2.46	23.41	2.87	206	-0.04	+0.18	3.26	4.02	7.41
**Mean 9 y/o**	**9.4 (±0.3)**	**+0.74 (±1.46)**	**23.31 (±0.93)**	**3.72 (±1.00)**	**195 (±8)**	**-0.04 (±0.07)**	**+0.13 (±0.03)**	**3.31 (±0.14)**	**3.87 (±0.21)**	**7.78 (±0.31)**
S11	11.0	+1.17	22.33	3.40	185	-0.06	+0.20	3.42	3.80	7.60
S03	11.6	-0.67	24.05	2.67	203	-0.01	+0.11	3.35	3.91	7.84
**Mean 11 y/o**	**11.3 (±0.5)**	**+0.25 (±1.30)**	**23.19 (±1.21)**	**3.04 (±0.52)**	**194 (±13)**	**-0.04 (±0.04)**	**+0.16 (±0.06)**	**3.39 (±0.05)**	**3.86 (±0.08)**	**7.72 (±0.17)**
**Mean all**	**8.2 (±1.3)**	**+0.61 (±1.14)**	**22.88 (±0.79)**	**3.92 (±0.95)**	**198 (±11)**	**-0.05 (±0.06)**	**+0.10 (±0.07)**	**3.40 (±0.14)**	**3.74 (±0.25)**	**7.73 (±0.26)**

^*a*^ Subjects with habitual correction with single vision spectacles.

**Fig. 2.**
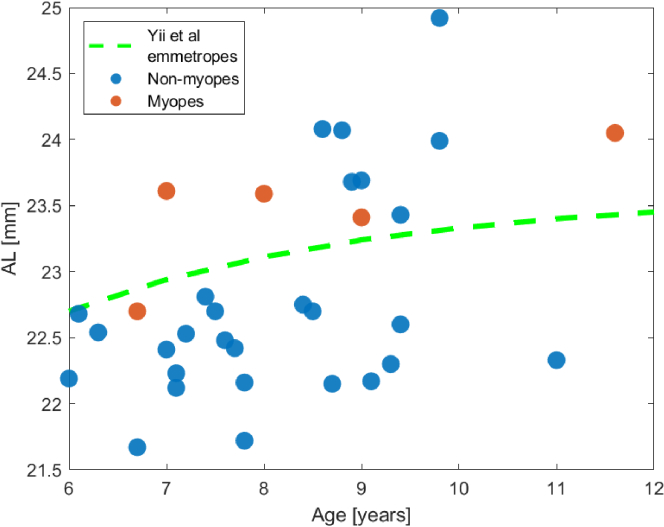
Relationship between age and axial length (AL) of each subject in our sample, with reference to an AL growth curve (green dashed line) based on emmetropes from different parts of the world [28]. Myopes (SER ≤
−
0.5 D) are marked as red dots.

To visualize the biometric representation of the sample in this study, Fig. 2 illustrates the relationship between AL and age in comparison to an AL growth curve of emmetropes (SER −
0.50 D to +1.25 D) [28]. In comparison to the emmetropic growth curve, most subjects in this study exhibited shorter AL for their age, which is reflected in the mean AL of 22.88±
0.79 mm as well as the mean SER of +0.61±
1.14 D of the sample as whole. As expected, myopic participants, represented by red dots in the scatter plot, had longer AL.

**Fig. 3.**
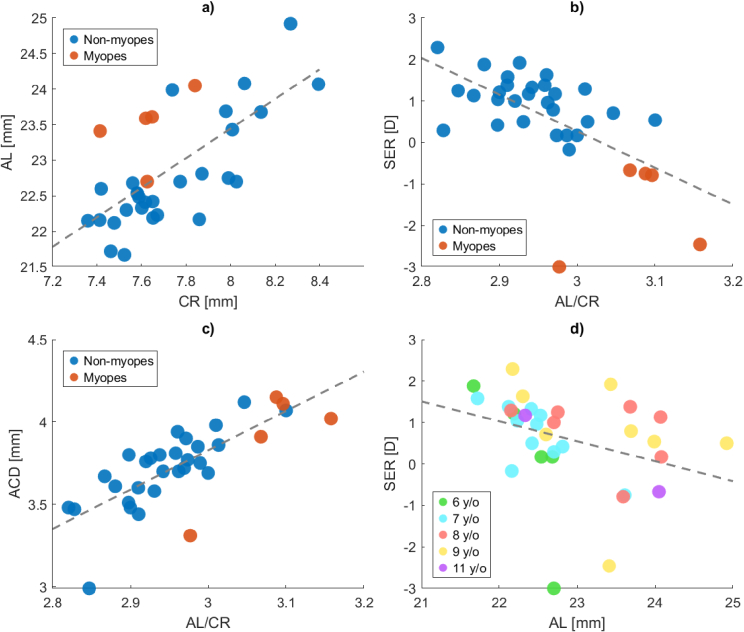
Correlations between some biometric parameters. Trends are illustrated with linear least square fits (dashed grey lines). a) Positive correlation between CR and AL ( ρ
=0.69 , 95% CI [0.45, 0.91], p<0.0001 ), myopes (SER ≤
−
0.5 D) are marked as red dots. b) Negative correlation between AL/CR and SER ( ρ
=−
0.61 , 95% CI [ −
0.35,−
0.87 ], p<0.001 ), myopes (SER ≤
−
0.5 D) are marked as red dots. c) Positive correlation between AL/CR and ACD ( ρ
=0.77 , 95% CI [0.57, 0.96], $p<10^{-6}$ ), myopes (SER ≤
−
0.5 D) are marked as red dots. d) Tendency toward a negative correlation between AL and SER ( ρ
=−
0.49 , 95% CI [ −
0.18,−
0.80 ], p<0.01 ), the subjects’ ages represented by different colors. AL = axial length, CR = corneal radius of curvature, ACD = anterior chamber depth, SER = spherical equivalent refraction.

Figures 3(a)-(c) show scatter plots of some of the biometric correlations that were significant (with Bonferroni correction), using Spearman correlation (see Table 2). As expected, it was found that CR and AL were positively correlated ( ρ
=0.69 , 95% CI [0.45, 0.91], p<0.0001 ) (Fig. 3(a)), which means that a flatter cornea is correlated with a longer axial length. We also see that the myopes had a longer AL in relation to CR than most of the non-myopes. This is further demonstrated as a significant negative correlation between SER and AL/CR ratio ( ρ
=−
0.61 , 95% CI [ −
0.35 , −
0.87 ], p<0.001 ) (see Fig. 3(b)). Figure 3(c) shows that the anterior chamber depth (ACD) and AL/CR ratio were found to be positively correlated ( ρ
=0.77 , 95% CI [0.57, 0.96], $p<10^{-6}$ ). ACD was also negatively correlated with LT ( ρ
=−
0.57 , 95% CI [ −
0.33 , −
0.80 ], p<0.001 ). This can be expected, since a thinner lens would result in a larger ACD.

**Table 2.** Correlation coefficients (Spearman’s ρ
 ) for biometric parameters. Note that with Bonferroni correction, only p<0.0014 is significant ( α
=0.05 ), indicated in bold in the table. AL = axial length, SER = spherical equivalent refraction, ACD = anterior chamber depth, LT = lens thickness, CR = corneal radius of curvature, LDR = radial lens decentration, LTA = lens tilt axial angle.

	AL	SER	Age	ACD	LT	CR	AL/CR	LDR	LTA
AL	1								
SER	− 0.49 ^*a*^	1							
Age	0.40	0.12	1						
ACD	0.24	− 0.26	0.38	1					
LT	− 0.28	0.03	− 0.33	− 0.57 ^*b*^	1				
CR	**0.69** ^*c*^	0	0.26	− 0.29	0.12	1			
AL/CR	0.33	− 0.61 ^*b*^	0.21	**0.77** ^*d*^	− 0.45 ^*a*^	− 0.38	1		
LDR	− 0.03	0.23	0.34	0.10	− 0.42	− 0.10	0.01	1	
LTA	− 0.17	0.25	− 0.24	− 0.13	− 0.01	0.11	− 0.37	0.42	1

^*a*^ p<0.01 ,

^*b*^ $p<10^{-3}$ ,

^*c*^ $p<10^{-4}$ ,

^*d*^ $p<10^{-6}$

Additionally, a trend toward a negative correlation ( ρ
=−
0.49 , 95% CI [ −
0.18 , −
0.80 ], p<0.01 ) was observed between AL and SER. Figure 3(d) presents this as a scatter plot in which the subjects’ ages are represented by different colors. No significant correlations were found between AL and age, nor between SER and age within our sample. Table 2 shows all calculated correlation coefficients between the different biometric parameters in our sample.

### 3.2. RPR with accommodation

On average, 708 wavefront images per subject were used for the wavefront analysis (minimum 262, maximum 1550). This corresponds to an average of 88 foveal-peripheral wavefront pairs per visual angle and accommodation target.

**Fig. 4.**
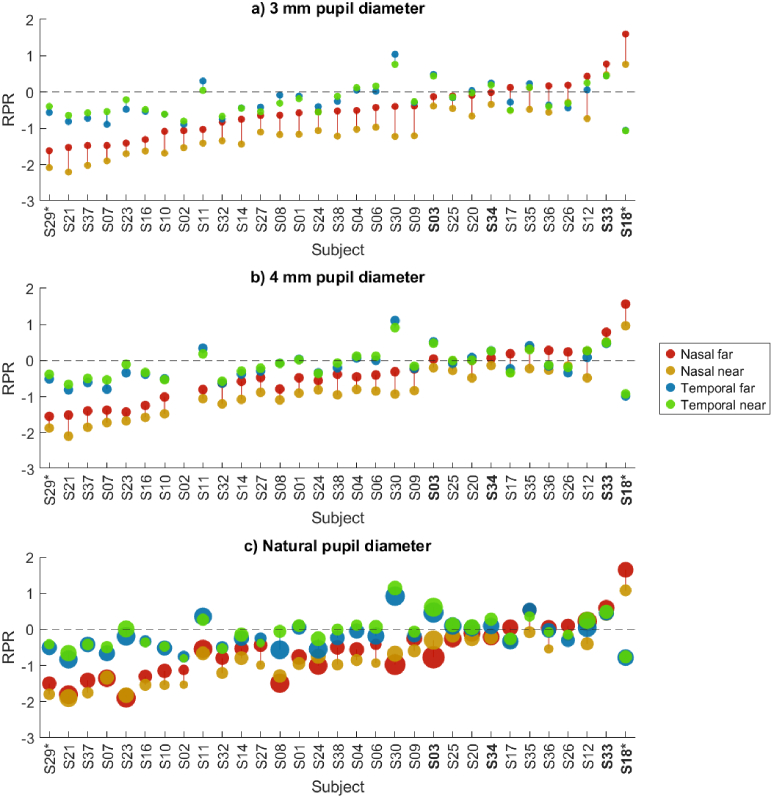
RPR for the $25^{\circ }$ nasal visual field and $25^{\circ }$ temporal visual field, measured for two accommodation targets (far at 0.22, near at 5 D). a) RPR for pupils scaled to 3 mm diameter. b) RPR for pupils scaled to 4 mm diameter. Note that for subject S02, the original pupil diameter was smaller than 4 mm, which is why no data is displayed for this pupil size. c) RPR for natural pupil diameters. Subjects are sorted according to the 3 mm nasal RPR at far. The widths of the dots are proportional to their corresponding pupil diameters, with the largest being 8.7 mm for subject S30 at nasal far with natural pupils. Subjects wearing their habitual correction during measurements are marked with *, and myopic subjects are marked with bold text.

Figure 4 shows RPR for the 31 subjects that had complete wavefront data (one myope (S31) and one non-myope (S19) missing compared to biometry data). RPR is shown for both levels of accommodation (far at 0.22 D, and near at 5 D) and for three pupil diameters (scaled to 3 mm, scaled to 4 mm, and natural/unscaled), calculated from the $2^{\text{nd}}$ -order Zernike coefficients. Myopic subjects are marked with bold, and subjects wearing habitual correction during wavefront measurements are marked with *. RPR is sorted ascendingly by nasal RPR at far (3 mm pupil diameter). From the figure, it is clear that nasal RPR had a larger intersubject variation than temporal RPR, ranging from approximately −
2 D to +2 D nasally compared to −
1 D to +1 D temporally. Furthermore, for most of the subjects, nasal RPR was more negative than temporal RPR. This asymmetry was found to be significant (using Bonferroni correction) for all pupil cases for the near measurements, and for natural pupils for the far measurements, see Table 3. We can also see that the myopic subjects were among the subjects with the most positive RPRs. Additionally, the intrasubject change of RPR with accommodation (i.e., the difference in RPR between far and near) was larger in the nasal visual field than in the temporal: near RPR was more negative than far RPR in the nasal visual field, but in the temporal visual field near RPR and far RPR were much more similar.

**Table 3.** RPR asymmetry (nasal RPR - temporal RPR). Values that are significant with Bonferroni correction (12 total RPR tests, leading to new significance level 0.05/12 = 0.004) are marked with bold. For the near measurements, there was a statistically significant asymmetry between nasal and temporal RPR, with nasal RPR being more negative, for all pupil sizes. For the far target, the asymmetry was only significant for natural pupils. Wilcoxon tests were used to test for significance.

Target	3 mm pupil	4 mm pupil	Natural pupil
Far	$-0.27\text{ D},\;p<10^{-2}$	$-0.21\text{ D},\;p<10^{-2}$	$-0.35\text{ D},\;p<10^{-3}$
Near	$-0.94\;\text{D},\;p<10^{-5}$	$-0.77\;\text{D},\;p<10^{-5}$	$-0.73\text{ D},\;p<10^{-5}$

**Fig. 5.**
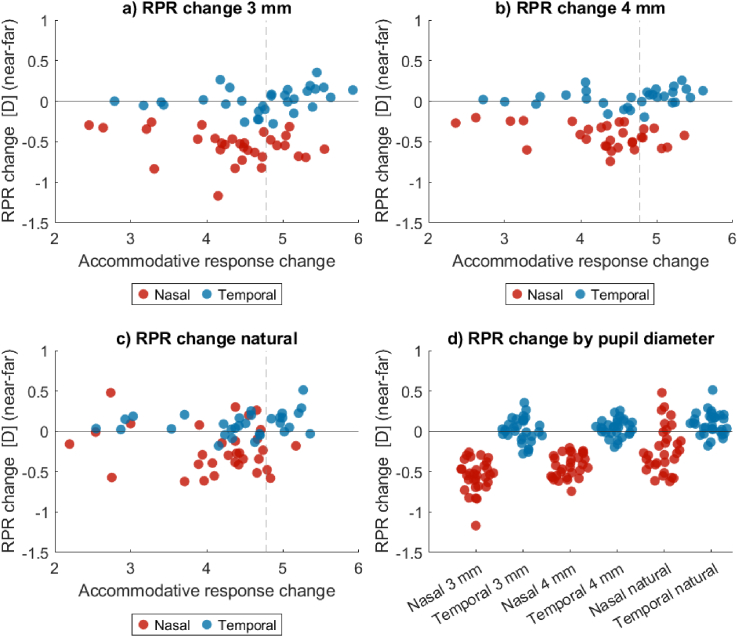
Change in RPR (difference between near and far RPR) with increased accommodation for all subjects. Note that the accommodative response change is dependent on pupil scaling. The dashed line indicates the expected accommodative response change of 5D−
0.22D=4.78D . a) RPR change for 3 mm pupils. b) RPR change for 4 mm pupils. c) RPR change for natural pupils. d) RPR change, grouped by pupil scaling and visual field.

Figure 5 explores RPR and accommodation further by showing the RPR *change* with accommodation on the y-axis, i.e., the difference in RPR for each subject between their near and far measurements, and the accommodative response change on the x-axis. Corresponding median changes and statistical significance (with Bonferroni correction) are found in Table 4. It is here clear that the RPR change with accommodation is not a result of pupil constriction with accommodation, since this shift in RPR is seen for both the scaled pupil cases and for natural pupils. For the 3 mm pupil case, nasal RPR became significantly more negative (myopic) with increased accommodation, but temporal RPR did not change significantly. For the 4 mm pupil case, the myopic shift in nasal RPR was still highly significant, and there was also a tendency toward a hypermetropic shift in temporal RPR. Only in the natural pupil case did both nasal and temporal RPR change significantly with accommodation, in different directions. The difference between how the nasal and temporal RPR were affected by increased accommodation seems to be larger with scaled pupils than with natural pupils, as seen most clearly in the comparison in Fig. 5(d). This shows that the change in RPR with accommodation is a direct result of the accommodation itself, and is not simply a result of accommodation-induced pupil constriction.

**Table 4.** Median RPR change with accommodation. Values that are significant with Bonferroni correction (12 total RPR tests, leading to new significance level 0.05/12 = 0.004) are marked with bold. We see that nasal RPR became significantly more negative with increased accommodation, for all pupil cases. Oppositely, temporal RPR became more positive, but only significantly so for natural pupils. Wilcoxon tests were used to test for significance.

Visual field	3 mm pupil	4 mm pupil	Natural pupil
Nasal	$-0.54\text{ D},\;p<10^{-9}$	$-0.43\text{ D},\;p<10^{-8}$	$-0.27\text{ D},\;p<10^{-3}$
Temporal	+0.004 D,p>0.05	+0.05 D,p<0.05	$+0.09\text{ D},\;p<10^{-3}$

From Fig. 5, we can also investigate the relationship between accommodative response change and RPR change. It would be expected that if RPR changes with accommodation, the amount of change would depend on the accommodative response change. However, the children that had a smaller accommodative response change (large lead at far or lag at near) still had similar RPR changes to those with larger accommodation changes.

Notably, some children only accommodated ≈
2.5 D more during the near measurements than the far, even though the accommodation demand had increased by 4.78 D. Unsurprisingly, the low-myopes who did not wear any habitual correction, were in this group. Due to their uncorrected myopia, they had an apparent “lead” at far, and they also had lag at near, resulting in the low accommodative response change.

For 3 mm pupils, there was a median foveal lag at near of 0.35 D for the nasal measurements, and a very slight lead of 0.01 D for the temporal measurements. This difference was probably due to increased tiredness during the nasal acquisitions, which were performed after the temporal ones.

### 3.3. Wavefront data correlations with biometry

Table 5 shows correlation coefficients (Spearman’s ρ
 ) between various image quality metrics and selected biometric parameters. The only statistically significant correlation (with Bonferroni correction) was nasal RPR at near with SER ( ρ
=−
0.56 , 95% CI [ −
0.25,−
0.87 ], $p<1.02\cdot 10^{-3}$ ). However, nasal RPR at far was also correlated with SER ( p<0.01 ), but not to the strong degree required by Bonferroni correction. Additionally, nasal RPR at far was also correlated with horizontal lens decentration ( p<0.01 ), but once again, not below the Bonferroni limit. No significant correlations were found between the biometric parameters and Strehl ratio.

**Table 5.** Correlation coefficients (Spearman’s ρ
 ) for image quality metrics vs. biometric parameters. Note that with Bonferroni correction, only $p<1.02\cdot 10^{-3}$ is significant ( α
=0.05 ). Image quality metrics were calculated for scaled wavefronts (3 mm pupil diameter). AL = axial length, SER = spherical equivalent refraction, LTX = horizontal lens tilt, LDX = horizontal lens decentration. RPR = relative peripheral refraction, Δ
 RPR = RPR change with accommodation, STR = Strehl ratio, STR acc. = STR corrected for foveal accommodation, STR target = STR corrected for target vergence, Lead/lag = difference between accommodation demand and response for near (average from nasal and temporal wavefront measurements).

	AL	Age	SER	LTX	LDX
RPR nasal far	0.31	0.23	− 0.49 ^*a*^	− 0.34	*0.50* ^*a*^
RPR temporal far	0.09	0.21	− 0.15	− 0.15	0.35
RPR nasal near	0.37	0.23	− 0.56 ^*b*^	-	-
RPR temporal near	0.06	0.21	− 0.08	-	-
Δ RPR nasal	0.11	− 0.08	− 0.18	-	-
Δ RPR temporal	− 0.01	− 0.08	0.30	-	-
STR nasal far acc.	− 0.12	− 0.09	0.02	-	-
STR temporal far acc.	− 0.04	− 0.26	0.10	-	-
STR nasal far target	0.00	− 0.10	− 0.10	-	-
STR temporal far target	− 0.14	− 0.01	0.28	-	-
STR nasal near acc.	− 0.23	− 0.25	0.05	-	-
STR temporal near acc.	0.07	− 0.15	0.11	-	-
STR nasal near target	0.28	0.21	− 0.35	-	-
STR temporal near target	0.12	0.18	− 0.25	-	-
Lead/lag	0.25	− 0.03	− 0.43	-	-

^*a*^ p<0.01 ,

^*b*^ $p<1.02\cdot 10^{-3}$ (Bonferroni limit)

## 4. Discussion

This study presents the baseline findings from the longitudinal Stockholm Myopia Study. We investigated uncycloplegic relative peripheral refraction (RPR) in the nasal and temporal visual fields for two levels of accommodation, in young schoolchildren recruited in Stockholm. This study employed a novel dual-angle wavefront aberrometer, measuring in the foveal and peripheral visual fields simultaneously, marking its first use in pediatric RPR measurements. Cycloplegic autorefraction and biometric parameters were also assessed.

### 4.1. Population and biometry

The children in our study represent a diverse range of ethnic backgrounds with approximately 45% relating to a foreign background other than Swedish. This reflects the large ethnic diversity of the population in Stockholm city, with 28.3% of 0- to 15-year-old children having a foreign background, either themselves being born in another country or having two parents born in another country than Sweden [29]. The children were recruited from schools, optometrists and eye clinics, with clear instructions to the children and their parents/guardians that the study aimed to investigate myopia development among schoolchildren. This may have attracted parents with a particular interest in myopia or those with scientific backgrounds, which could have influenced the composition of the study sample.

The refractive state of the children was defined as myopic for SER ≤
−
0.5 D, in accordance with Yii et al. [28]. Consequently, 5 of 33 children were myopic, which is in line with the results in previous Scandinavian studies. We chose not to define emmetropia and hypermetropia, as the children in our sample are young enough to still be undergoing emmetropization, with AL still growing and SER changing [30].

The biometric findings are reported to describe the population sample of the study, and were found to align quite well with previous Scandinavian studies [31,32]. The mean AL in this study was 22.88 ( ±
 0.79) mm. Compared to the world average AL for emmetropes [28], most subjects in the current study exhibited a shorter AL in relation to their age, which could be due to that we did not differentiate between emmetropes and hypermetropes. It is well known that longer AL is correlated with lower SER and myopia [33–36], and as expected, the myopic participants had longer AL. The mean CR was 7.73 ±
 0.26 mm and as expected, CR and AL in our study were found to be positively correlated, showing that a flatter cornea was correlated with a longer axial length. The AL/CR ratio has been shown to correlate with different refraction groups, with myopes having a higher AL/CR ratio and hypermetropes having a lower AL/CR ratio [36,37]. Our study agrees with these previous findings, as a significant negative correlation between SER and AL/CR ratio was found. The positive correlation between ACD and AL/CR ratio also agrees with findings in previous studies [36].

### 4.2. RPR and image quality

We measured RPR with a custom-built open-field-of-view dual-angle wavefront aberrometer. The benefit of using this instrument is that we get simultaneous measurements of foveal and peripheral image quality, allowing us to calculate RPR accurately in a natural binocular setting for multiple levels of accommodation. As we wanted to measure the everyday RPR that the eye experiences, the children who wore habitual correction were instructed to wear their correction during the wavefront measurements. Since spectacles have been shown to alter RPR [38–40], it is not unexpected that these children turned out to have among the largest absolute RPRs of our study sample.

RPR was calculated only from the $2^{\text{nd}}$ -order Zernike coefficients. This meant that RPR was dependent on pupil scaling, which is why we have presented results both for natural pupils and two sets of scaled pupils. Another option would have been to use Seidel refraction, which only considers the paraxial refraction by also incorporating higher-order Zernike terms. However, using only $2^{\text{nd}}$ -order terms allows for comparisons with previous studies.

#### 4.2.1. RPR asymmetry

Generally, RPR was larger and more negative in the nasal visual field than in the temporal, and correspondingly, many of the children had asymmetric RPR in the horizontal visual field, especially with increased accommodation. This asymmetry could be explained by the difference between the visual axis and the optical axis of the eye, which is typically around ${\mathrm{3–5}}^{\circ }$ [41], with the optical axis being toward the temporal side of the visual field. This would mean that relative to the optical axis, we have measured at ${\mathrm{28–30}}^{\circ }$ nasally, and ${\mathrm{20–22}}^{\circ }$ temporally. Furthermore, as the eye is stiffer around the optical nerve, we could expect that the eye would grow slower around the optical nerve head, which is in the temporal visual field, than in other parts of the retina. This would, in turn, result in a longer eye in the nasal visual field than in the temporal, which could also explain the asymmetry with the more myopic RPR in the nasal visual field. In our study, we also found a weak correlation between nasal RPR at far and horizontal lens decentration, yielding an additional factor for asymmetry.

Previous studies have also reported asymmetries in horizontal RPR, at least for some subgroups, such as for myopic children [15,42–44], and for emmetropic children [44,45]. However, these asymmetries are not consistent between studies, neither in terms of subgroups, nor in direction. Furthermore, these studies all used cycloplegic autorefraction to obtain RPR, and the use of cycloplegia has been demonstrated to alter RPR compared to uncyclopleged measurements in adults [18]. A recent study by Vera-Diaz et al. [46] measured RPR at far for children 6–9 years old without cycloplegia. They found that children with low risk for myopia had more hypermetropic RPR in the temporal visual field than in the nasal, but the trend reversed for myopic children. In our study, no subgroup analysis was made for RPR between the refractive groups, due to the small sample size for myopes. However, as most of the children had a more hypermetropic/less myopic RPR temporally than nasally, it is likely that they are at low risk to develop myopia.

#### 4.2.2. RPR change with accommodation

In the present study, we have measured RPR for two levels of accommodation: 0.22 D and 5 D, finding negative shifts with accommodation in the ${\mathrm{25}}^{\circ }$ nasal visual field, and a possible slight positive shift in the ${\mathrm{25}}^{\circ }$ temporal visual field. To our knowledge, the present study is the first to measure RPR with accommodation in children. On adult emmetropes, some previous studies have found negative shifts in both the ${\mathrm{20–30}}^{\circ }$ nasal and temporal visual fields [47–49], others positive shifts [50–52], but none has reported similar trends to ours with clear negative shifts nasally and no or small positive shifts temporally. Neither have any studies on adult myopes; one has reported only positive shifts [50], one only negative [53], and two more positive nasally, but more negative temporally [47,48]. However, these studies used different accommodation levels, and a study by Queirós et al. found that the sign of RPR shifts could depend on the level of accommodation [54]. Therefore, more studies are needed, on both adults and children, preferably using more than two accommodation levels, to fully understand the effect of accommodation on RPR. Additionally, only one previous study, by Romashchenko et al. [55], has measured foveal and peripheral refraction simultaneously. If refraction is not measured simultaneously, there is a risk of errors in measured refraction between the foveal and peripheral measurements, due to changes in accommodation and pupil size.

#### 4.2.3. Impact on myopia progression?

The correlation between RPR and SER has been well established [56–58], with myopia generally being coupled with hypermetropic RPR. We also found this link in our study population, for RPR in the nasal visual field, both at far and near. However, there is conflicting evidence on whether a more hypermetropic RPR is caused by the axial elongation in myopia, or if RPR is a driving factor in myopia development [58]. Animal studies generally point in the direction of RPR playing a role in refractive development [7–10,58–60]. Studies on humans have shown varying results, with some finding causative links [45,61], others not [15,43,44,62–64]. Furthermore, progressive spectacles that cause a myopic shift in RPR has been shown to slow myopia progression [65–67], and many new myopia control treatments use multifocal optics with positive add power, aiming to focus part of the light in front of the peripheral retina [13,14,68,69]. However, recently, a new type of optical myopia control that uses scattering instead of multifocality has also been shown to be successful in controlling myopia progression [70], as well as a multifocal design with negative add power instead of positive [71]. As new research on multifocal designs has shown that they generally reduce the through-focus peripheral image quality, and do not necessarily move the sharpest image more in front of the retina [17], these newer designs have raised further questions regarding the working mechanism of optical myopia control [16]. In the present study, we therefore explored not only RPR, but also peripheral Strehl ratio, and their correlations with SER. However, no clear correlation was found between the peripheral Strehl ratio and SER or AL for these young schoolchildren.

During the coming part of the Stockholm Myopia Study, we will continue to monitor the ocular growth and peripheral image quality yearly. This will allow us to analyze how the image quality is related to emmetropization and myopia development longitudinally. Compared to previous longitudinal RPR research, our RPR measurements are performed with our dual-angle wavefront aberrometer, which allows us to capture the natural RPR without cycloplegia, both for distance viewing and near work, providing a more complete picture of the RPR and peripheral image quality.

#### 4.2.4. Implications for near work

We measured RPR during accommodation because both RPR and near work are often related with myopia progression and control. Since hypermetropic RPR is connected with increased ocular growth, we would have expected that near work would induce a more hypermetropic RPR. However, we found the opposite trend, at least for the nasal visual field. In the temporal visual field, RPR was largely unaffected or became slightly more positive, depending on pupil scaling. This could indicate that the temporal visual field plays a larger role in myopia development than the nasal visual field, or that the myopic blur in the nasal visual field during accommodation to a smaller object (like a mobile phone) becomes too large to act as a stop signal for further ocular growth (similar to form deprivation). On the other hand, it could also mean that other factors (such as accommodation lag) are the primary reasons that near work is connected with myopia development.

## 5. Conclusion

This pilot study utilizes a unique research instrument together with state-of-the-art clinical instrumentation to evaluate the peripheral image quality during accommodation in 31 children. We found an asymmetry in RPR, with more negative refraction in the nasal visual field than in the temporal, for the majority of the children. The asymmetry remained, or even increased, with accommodation. The RPR in the nasal visual field was correlated with SER for +5 D of accommodation, with less negative RPR for the myopic children. The presented data is the first RPR measurements with accommodation in children, and forms the baseline measurements of the Stockholm Myopia Study. In the Stockholm Myopia Study, children are followed longitudinally, with follow-up measurements once per year. This will enable investigations on how the peripheral image quality, for both far and near vision, develops over time and how it is correlated with myopia development.

## Data Availability

Data underlying the results presented in this paper are not publicly available at this time but may be obtained from the authors upon reasonable request.
